# Modulating bacterial and gut mucosal interactions with engineered biofilm matrix proteins

**DOI:** 10.1038/s41598-018-21834-8

**Published:** 2018-02-22

**Authors:** Anna M. Duraj-Thatte, Pichet Praveschotinunt, Trevor R. Nash, Frederick R. Ward, Peter Q. Nguyen, Neel S. Joshi

**Affiliations:** 1 Wyss Institute for Biologically Inspired Engineering, Harvard University, Boston, MA United States; 2 John A. Paulson School of Engineering and Applied Sciences, Harvard University, Cambridge, MA United States

**Keywords:** Applied microbiology, Inflammatory bowel disease, Biomedical engineering

## Abstract

Extracellular appendages play a significant role in mediating communication between bacteria and their host. Curli fibers are a class of bacterial fimbria that is highly amenable to engineering. We demonstrate the use of engineered curli fibers to rationally program interactions between bacteria and components of the mucosal epithelium. Commensal *E. coli* strains were engineered to produce recombinant curli fibers fused to the trefoil family of human cytokines. Biofilms formed from these strains bound more mucins than those producing wild-type curli fibers, and modulated mucin rheology as well. When treated with bacteria producing the curli-trefoil fusions mammalian cells behaved identically in terms of their migration behavior as when they were treated with the corresponding soluble trefoil factors. Overall, this demonstrates the potential utility of curli fibers as a scaffold for the display of bioactive domains and an untapped approach to rationally modulating host-microbe interactions using bacterial matrix proteins.

## Introduction

The mucosal surfaces of the gastrointestinal (GI) tract serve several important protective functions, including lubricating the epithelium, decreasing the shear forces experienced by its constituent cells, and trapping debris and bacteria^1^. They do this, in part, through the formation of a microporous gel-like mucus layer composed of proteoglycans (i.e. mucins) that are secreted by the epithelial cells. As the outermost layer of the GI tract, mucus plays a critical role in mediating the interaction between bacteria and the host, especially in the colon, where the highest concentrations of microbes are found. It does this by providing a physical barrier to separate bacteria from the epithelial cells, but also by regulating bacterial growth rate and preventing bacterial aggregation^2^. Defects in the mucus layer that are the result of dysregulation of mucin production or excessive degradation of mucins can have dire physiological consequences, including compromising intestinal barrier function and chronic inflammation that is caused or exacerbated by bacterial penetration into the epithelium^3^.

Naturally occurring pathogens and commensals employ several strategies to persist within the mucus layer, for example by feeding off the mucin glycans^4^. Another commonly found strategy is the surface display of adhesins and extracellular appendages with specific mucin binding capabilities^5^. Indeed, in some cases, deletions of individual genes encoding such adhesive proteins can abolish the colonization ability of a commensal microbe, or the pathogenicity of an invasive species, highlighting their importance for organismal fitness in the gut environment^5^. The display of engineered chimeric adhesins on the surface of non-pathogenic *E. coli* has even been demonstrated to enhance their localization to tumors when injected intravenously^6^. Furthermore, some adhesins can influence host biology directly through immunomodulation, and binding to innate immune receptors on the host cell surface^7,8^.

As a result of the proliferation of basic science aimed at understanding host-microbe interactions, there has been a corresponding swell of interest in engineering microbes for diagnostic and therapeutic purposes. Many of these efforts attempt to exploit features of commensal and probiotic microbes such that they can serve as genetically programmable sensors and drug delivery devices^9,10^. This is usually accomplished through the engineered secretion of soluble molecular factors that can directly influence host biological processes^11,12^. Although this overall approach continues to show promise as a therapeutic strategy, there are also challenges, one of which is achieving a sufficiently high local concentration of therapeutic molecule at the site of disease.

**Figure 1:**
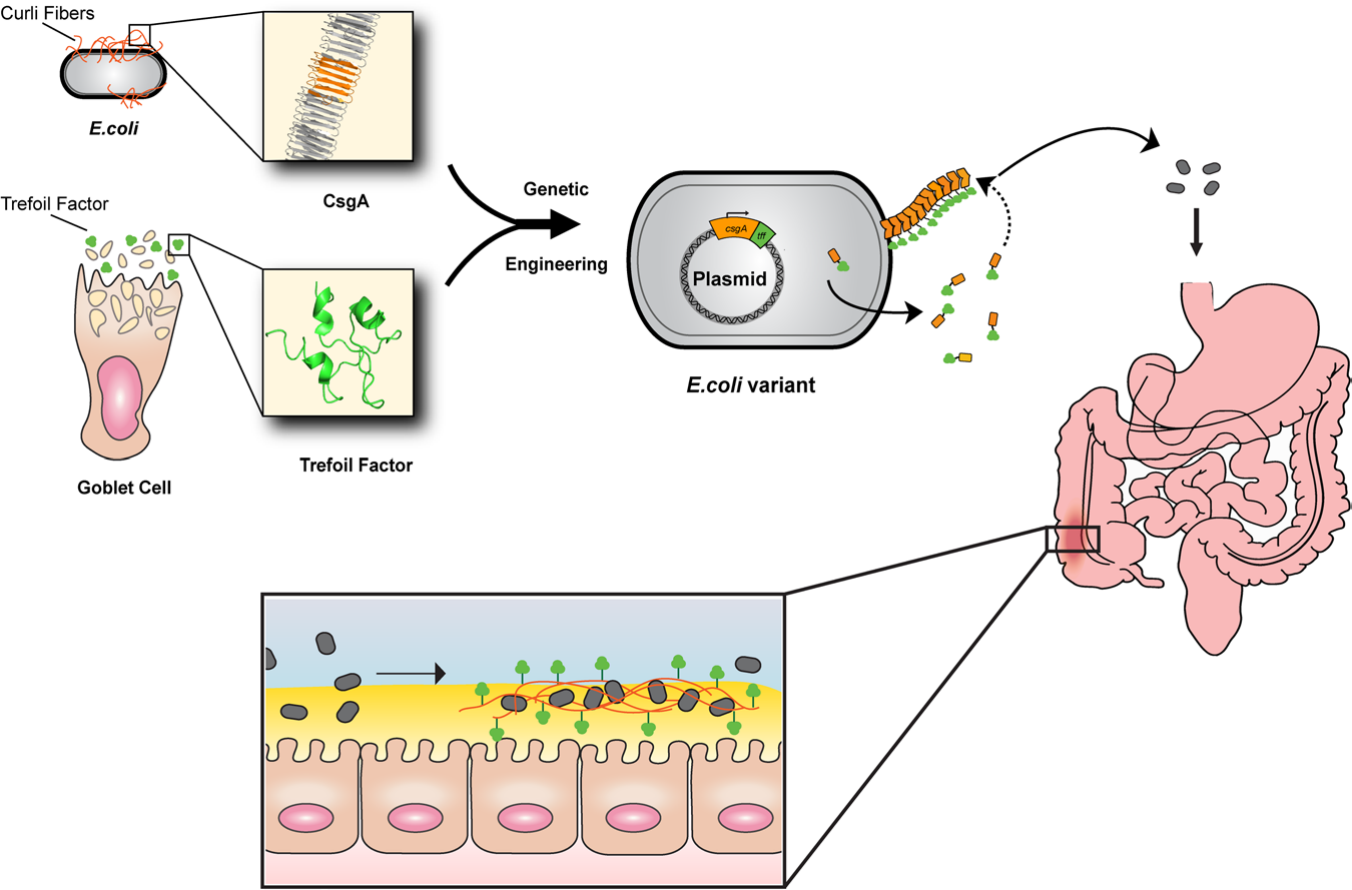
Repurposing commensal *E. coli* curli fiber proteins through TFF display (**A**). Schematic overview of reprogrammed *E. coli* curli nanofibers. CsgA, the main proteinaceous component of *E. coli* biofilm matrix, assembles into extracellular amyloid fibers after secretion in the monomeric form (top, inset shows one CsgA monomer highlighted in orange). Trefoil factors, like TFF3 (PDB ID: 1PE3, green), are cytokines secreted by mucus producing cells., *E. coli* was engineered to display the trefoil factors as genetic fusions to the C-terminus of CsgA. CsgA-TFF chimeras are self-assembled into nanofibers extracellularly (**B**), where they can modulate interactions with mucosal epithelium tissues (**C**).

We propose an alternative strategy to the secretion of soluble therapeutic factors, in the form of reprogramming biofilm matrix proteins. Here, we report our efforts to repurpose curli fibers of *E. coli*, the best studied and most frequently used organism for engineered probiotic efforts, to act as a display system for bioactive domains^12,13^. The result of this effort is an engineered bacterium that produces a self-assembled matrix *in situ* with a programmable function (Fig. 1). We show that we can program such a matrix to display specific protein domains that function simultaneously to enhance adhesion to mucins and epithelial cell surfaces, and modify cell behavior.

## Results

### TFF-fused curli fibers are produced, secreted, and assembled by commensal *E. coli*

Curli is a functional amyloid that is produced by *E. coli* and other *Enterobacteriaceae* during biofilm formation^14^. We have previously shown that the main structural component of curli fibers, CsgA, can be fused to a range of heterologous domains without abolishing its ability to be secreted and assembled extracellularly^15^. In order to enhance the interaction of curli nanofibers with the gut mucosa, we genetically fused CsgA to the trefoil factors (TFF1, TFF2, and TFF3), a family of human cytokines that are secreted by mucus-producing cells and goblet cells into the gut lumen and contribute to the maintenance of homeostasis (Figure S1). The entire TFF sequences were fused to the C-terminus of CsgA via a flexible linker domain in a manner that is very similar to previously published CsgA fusion proteins^16^. All three TFFs exhibit specific mucin binding activities^14,17,18^, enabling them to increase the viscosity of co-secreted mucins, possibly enhancing barrier function locally^19,20^. In addition to their biophysical effects, they also help maintain barrier function by promoting epithelial restitution and reinforcing tight junctions, among other mechanisms^21,22^. This has led to their investigation as therapeutics in the treatment of IBD, although delivery remains a challenge^23^. Furthermore, they range in size from 59–106 amino acids, making them compatible with the constraints of the curli secretion system.

**Figure 2:**
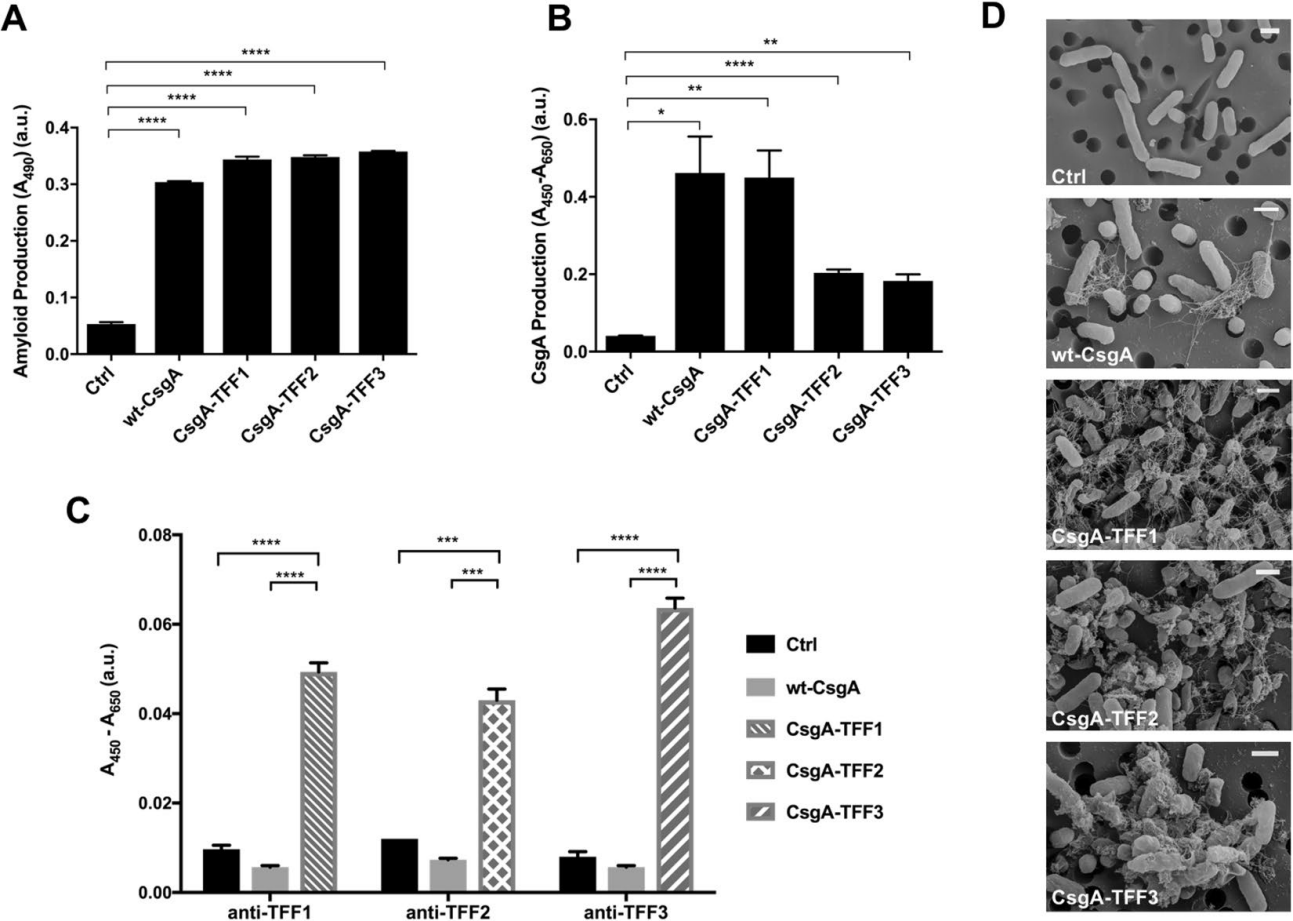
CsgA-trefoil factor fusions retain secretion and self assembly functionality. Congo Red staining assays (**A**), and whole-cell filtration ELISA assays with anti-CsgA antibody detection (**B**) confirm the formation of recombinant curli amyloids. Ctrl indicates cells containing a plasmid with no CsgA encoding gene. (**C**) Whole cell filtration ELISA was also used to detect each of the displayed trefoil factors, with the corresponding anti-TFF antibodies. (**D**) SEM images of *E. coli* PHL628-ΔcsgA strain transformed with plasmids encoding for no curli (Ctrl), wt-CsgA, and curli with displayed trefoil factors (CsgA-TFF1, CsgA-TFF2, CsgA-TFF3). All scale bars are 1 µm. Ordinary one way ANOVA with Dunnett’s multiple comparison test n = 3, *P < 0.05, **P < 0.01, ***P < 0.001, ****P < 0.0001.

We envisioned that the amyloidogenic fusion proteins would self assemble into a nanofibrous matrix with mucoadhesive and epithelial wound healing properties (Fig. 1). Accordingly, we prepared plasmids encoding CsgA-TFF fusions under the control of an isopropyl β-D-1-thiogalactopyranoside (IPTG) inducible promoter. These were transformed into a commensal *E. coli* strain (PHL628-Δ*csgA*) suited to curli overproduction^24,25^. A Congo Red (CR) dye binding assay, commonly used to test for the presence of amyloids, suggested that all three fusions could be successfully produced and assembled with comparable efficiency to the wild-type CsgA protein (Figs 2A and S2). We also performed a whole-cell filtration ELISA using antibodies against CsgA, which confirmed that the fusion proteins were successfully secreted and assembled extracellularly (Fig. 2B). We did observe a decrease in signal for the TFF2 and TFF3 constructs, which could reflect a legitimate difference in production, but could also reflect an altered affinity of these constructs for the anti-CsgA antibody, or accessibility of the TFF epitopes due to extensive fiber aggregation. Similar ELISA assays using antibodies against the individual trefoil factors confirmed the presence of the appropriate TFF for each variant (Fig. 2C). Scanning electron microscopy (SEM) analysis confirmed that all three fusion proteins assembled into nanofibrous structures typical of curli fibers, although CsgA-TFF2 and CsgA-TFF3 fibers did appear to aggregate more than those of wt-CsgA and CsgA-TFF1 (Fig. 2D).

### Engineered curli matrices bind to mucins and modify their biophysical properties

**Figure 3:**
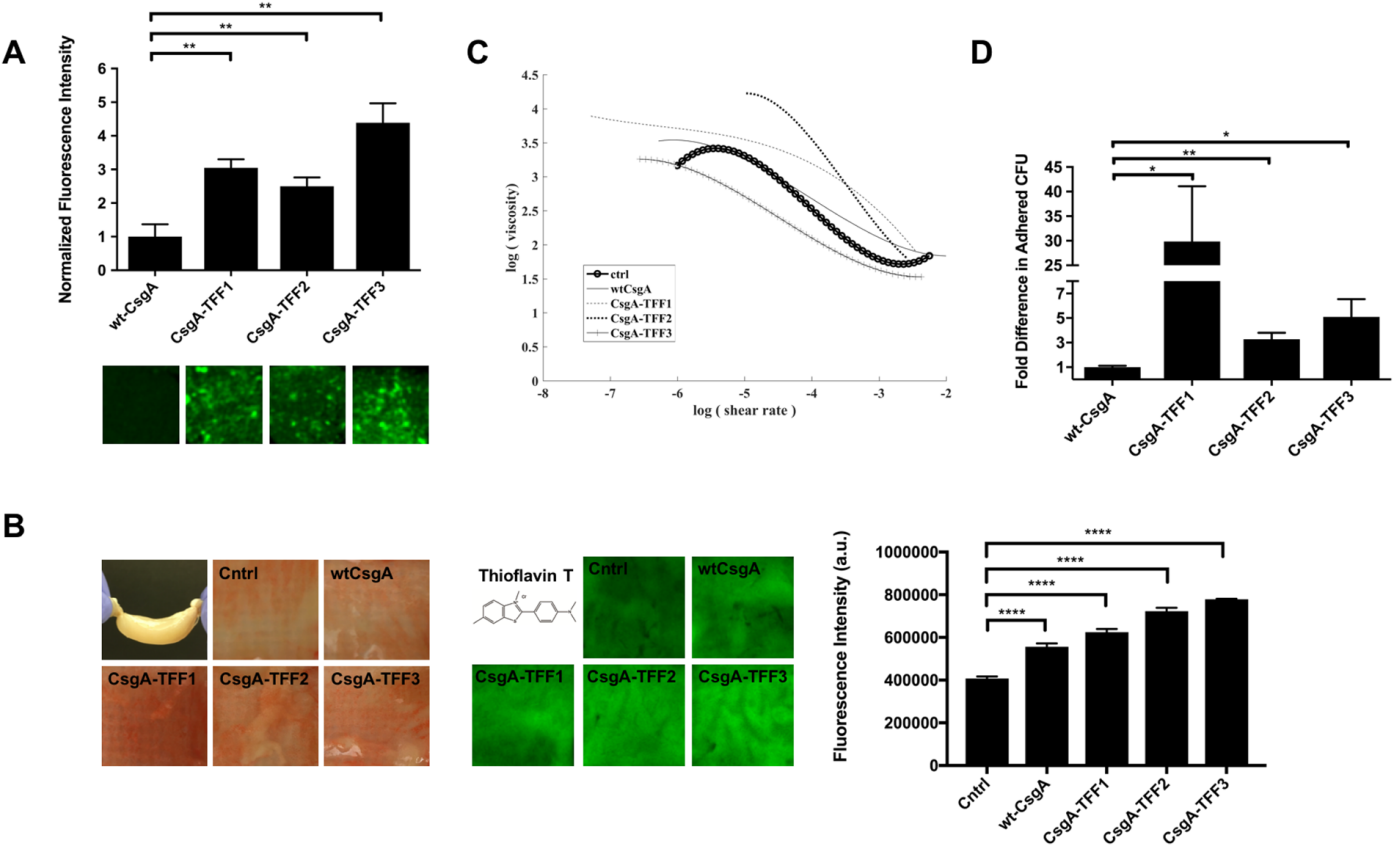
Engineered curli matrix enhances *E. coli* adhesion to gut mucosa. (**A**) Anti-Muc-2 detection of mucins after incubation on top of filtered biofilms producing curli-TFF fusions, (**B**) Congo Red and Thioflavin T staining of goat colon after incubation with engineered curliated E. coli variants. (**C**) Viscosity vs. shear rate analysis for mucins combined with curliated bacterial cultures. Ctrl indicates cells containing a plasmid with no CsgA encoding gene. (**D**) Bacterial adhesion to Caco-2 cell monolayers. Ordinary one way ANOVA with Dunnett’s multiple comparison test n = 10, *P < 0.05, **P < 0.01, ***P < 0.001, ****P < 0.0001.

Given that commensal forms of *E. coli* reside mostly in the outer colonic mucus layer^26^, we sought to evaluate the effects of TFF display on the interaction of the engineered *E. coli* with mucins. Soluble endogenous trefoil factors have a characteristic triple-loop structure that is held together by intramolecular disulfide bonds. The TFFs are known to bind to mucin proteins through a combination of nonspecific hydrophobic interactions and disulfide exchange with the cysteine-rich von Willebrand factor (VWF) domains common to all mucins^17^. To determine the mucin binding activity of curli-displayed TFFs, we first assessed the binding of type II porcine gastric mucins (a common model system for gastrointestinal mucins) to filtered biofilms. As expected, all of the CsgA-TFF fusions enhanced binding of the biofilms to mucins significantly, with CsgA-TFF3 binding to more than 4 times the amount of mucin as wt-CsgA (Fig. 3A). In order to evaluate the binding activity of the engineered *E. coli* strains with mucins in a more physiologically relevant model, we developed an *ex vivo* assay with a section of goat intestine (i.e. the “sausage” assay, Fig. 3B). After incubating the engineered strains in fresh tubular sections of colon and gently washing to remove unbound bacteria, we found that all three TFF fusions led to higher CR staining intensity on the mucosal surface compared to bacteria producing wt-CsgA or no CsgA. We also stained the intestine samples with thioflavin T (ThT), which, along with CR, is commonly used as a stain for amyloid deposits in histopathology of animal tissues. Upon binding to amyloids, ThT fluorescence intensity increases significantly. Although there was a large amount of background signal from the tissue itself, making direct comparison of the CR and ThT staining difficult, samples treated with curli-producing bacteria showed significantly more staining compared to the tissue alone. Furthermore, samples treated with bacteria producing curli-fused TFFs bound more ThT than those treated with bacteria producing wild-type CsgA.

In addition to binding to mucins, soluble TFFs have been shown to modulate the viscoelastic properties of mucin^20,27^. For example, TFF2 increases the viscosity of porcine gastric mucins. In the same study TFF1 and TFF3 did not increase the viscosity of the mucins, but instead formed small complexes with the mucins that could be easily visualized with optical microscopy. We sought to demonstrate similar principles of biophysical modification for the curli-displayed TFFs. Accordingly, bacteria expressing CsgA-TFF fusions were combined with type II mucins from porcine stomach and the mixture subjected to rheological analysis. While bacteria expressing wt-CsgA appeared to have only a minor effect on mucin viscosity as a function of shear rate, CsgA-TFF1 and CsgA-TFF2 fusions exhibited ~3-fold and ~10-fold increases in viscosity at a shear rate of 10^−5^ s^−1^, respectively (Fig. 3C).

### Engineered curli matrix enhances bacterial binding to mammalian cell surfaces

In addition to their mucin binding activity, bacterial adhesins also play a role in signaling through direct contact with epithelial cells^28^. Therefore, we wanted to determine how TFF display affects the interaction of engineered *E. coli* with the epithelial cell surface. There are some known binding interactions between TFFs and various epithelial cell lines^29,30^, and specific binding interactions between TFF2 and CXCR4 have also been reported^31^. We performed an adhesion assay by co-incubating engineered PHL628-Δ*csgA* with Caco-2 cell monolayers for 2 hours, followed by washing to remove non-adherent bacteria. The number of remaining bacterial cells was determined by CFU counting on antibiotic selective plates after lysis of the mammalian cells. All three displayed TFFs significantly enhanced bacterial binding to the cell surface compared to wild-type curli fibers, with TFF1 display increasing bacterial adhesion by almost 30-fold (Fig. 3D). In order to confirm that bacterial cell invasion did not lead to an over-estimation of adhesion, we also performed an invasion assay with Caco-2 cell monolayers. Two hours after inoculation of the Caco-2 cells with induced bacterial cultures, extracellular bacteria were killed with a non-cell permeable antibiotic (gentamicin). After lysis of the mammalian cells, we did not observe any CFU of the engineered bacteria on selective agar plates, confirming that curli fiber expression increased cell adhesion without inducing an invasive phenotype.

### Curli-bound trefoil factors maintain their signaling bioactivity

**Figure 4:**
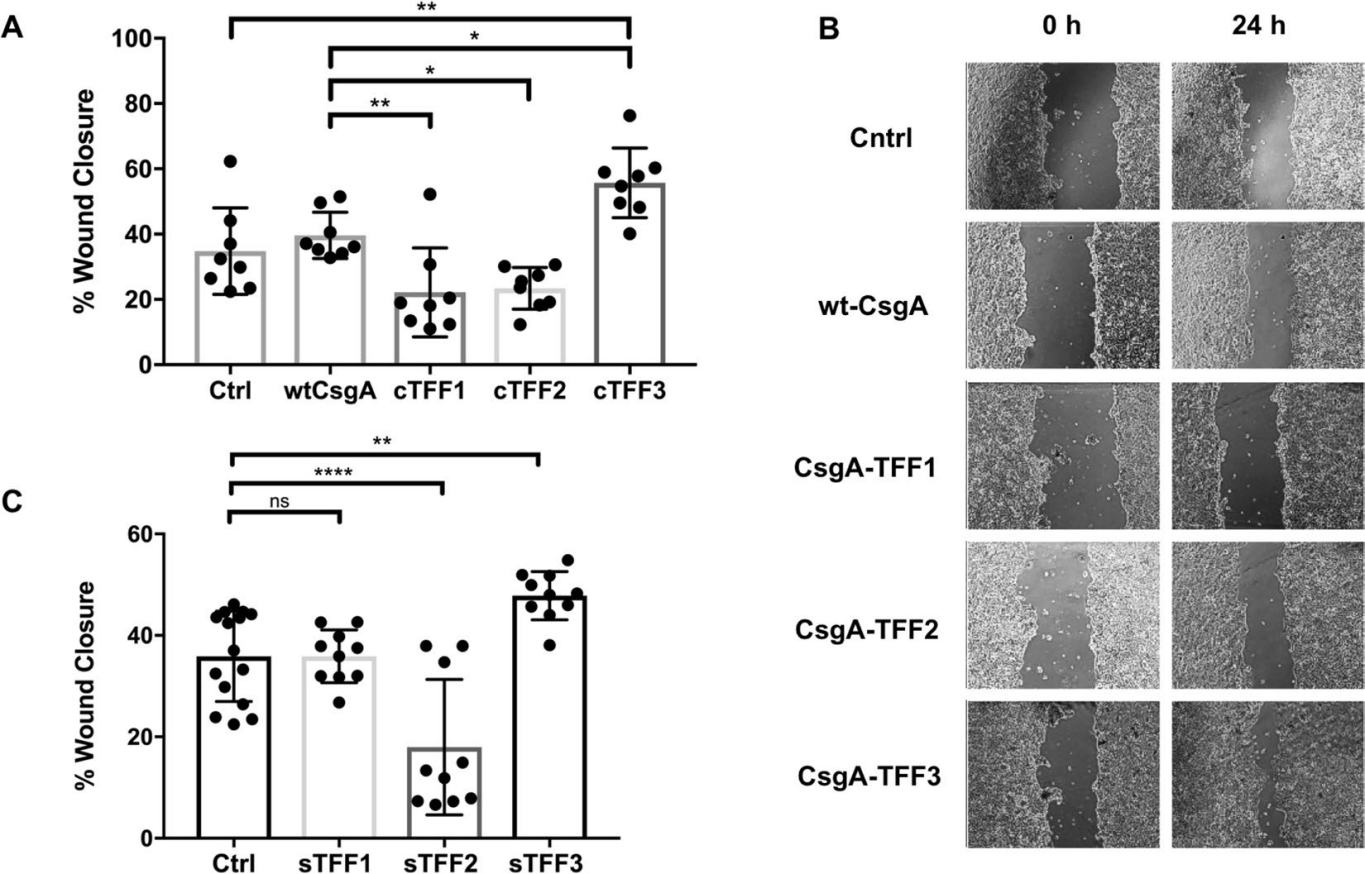
Bioactivity of displayed trefoil factors on the curli matrix (**A**). Percent cell monolayer defect closure for Caco-2 cell monolayers during incubation with various soluble semi-purified curli variants. Bars represent mean % defect closure after 24 hours. Ctrl indicates scratched Caco-2 monolayers with no treatment. (**B**) Representative images of defects and their progressive closure after 24 hours in the presence of various curli fiber variants. (**C**) Corresponding cell migration assay with soluble trefoil factors. Ordinary one way Anova n = 10, **P < 0.01, ***P < 0.001 Ordinary one way ANOVA with Dunnett’s multiple comparison test n = 10, *P < 0.05, **P < 0.01, ***P < 0.001, ****P < 0.0001.

Previous studies have shown that TFFs play important roles in regulating biological responses to GI inflammation and injury. The mechanisms by which they regulate this response are numerous and not completely understood, but all three TFFs are upregulated in response to injury and promote cell migration to restore barrier function to denuded mucosal lesions^32^. In order to determine if the curli-bound TFFs maintained their signaling bioactivity, we performed an *in vitro* cell migration assay. This type of assay is difficult to perform in the presence of bacteria because prolonged co-culture is not possible under standard conditions. Therefore, we used semi-purified curli fibers prepared through a filtration protocol recently developed in our lab^16^. Following induction of a defect in a confluent Caco-2 monolayer by scratching with a pipet tip, the cells were co-incubated with semi-purified curli fibers overnight. Defect closure induced by engineered curli fibers was compared to that induced by the corresponding soluble TFFs. Surprisingly, we found that, of the soluble TFFs, only TFF3 enhanced defect closure, while soluble TFF2 actually decreased the defect closure rate and TFF1 did not have a significant effect (Fig. 4C). TFF3 maintained its ability to promote cell migration when bound to curli fibers (Fig. 4A,B). TFF1 and TFF2 did not show a difference in defect closure compared to untreated controls, but showed a slight decrease compared to curli fibers composed of wt-CsgA.

## Discussion

Overall, this work demonstrates the first example of engineered curli fibers undergoing programmed interaction with biological and tissue surfaces through the display of bioactive domains. Overall, it represents the early stages of a new approach to reprogram host-microbe interactions. The ability to fuse large (>50 amino acid) polypeptide domains, including whole proteins on CsgA without abolishing secretion and self-assembly capabilities has been shown^33^. However, this work demonstrates that even domains like TFF2, which contains seven internal disulfide bonds, can be successfully displayed using this approach. The two primary functions of TFFs that make them potentially useful for treating inflammation in the GI tract^23^ – binding to mucins to enhance their protective properties, and promoting epithelial wound healing – are preserved *in vitro* even while the TFFs are bound to curli fibers. The platform we report here offers the opportunity to present TFFs and other bioactive domains as a multivalent array in a cohesive material format. Indeed, the versatility of the curli biosynthetic machinery suggests that this approach could be easily adapted for the display of other therapeutic domains. Curli-based materials could even be programmed with multiple bioactive functions simultaneously, as we have shown previously^15,34^. Current efforts in our group are focused on implementing this overall approach in commensal strains suitable for oral delivery with the long-term goal of engineering a therapeutic microbe capable of creating a responsive, resident biomaterial inside the GI tract with prescribed properties. This material, which could be pre-programmed to exhibit specific features, could then be administered orally to treat a range of diseases.

## Methods

### Cell Strains and Plasmids

Genes encoding for the CsgA-TFF1-3 fusion proteins were synthesized (Integrated DNA Technologies) and cloned by overlap extension into pBbE1a vectors by using Gibson Assembly^35^. All experiments involving engineered curli expression were performed in a *csgA* deletion strain (MG1655, *malA:kan*^*r*^ *ompR234* Δ*csgA*, a.k.a. PHL628-Δ*csgA*) that was kindly provided by the Hay Laboratory (Cornell University).

### Curli nanofiber expression

To express curli nanofibers, PHL628-∆*csgA* was transformed with pBbE1a plasmids encoding for wt-CsgA, CsgA-TFF1, CsgA-TFF2, or CsgA-TFF3, or a control plasmid without any CsgA encoding gene. Transformed cells were streaked onto fresh lysogeny broth (LB) agar plates supplemented with 100 μg/mL carbenicillin and were grown overnight at 37 °C. A single colony was picked, inoculated in LB medium containing 100 μg/mL carbenicillin and incubated overnight at 37 °C. The overnight culture was diluted 1:100 in fresh LB medium with 100 μg/mL carbenicillin grown and the protein expression was carried out overnight at 37 °C.

### Quantitative Congo Red (CR) binding assays

Congo Red (CR) binding assays were adapted from previously described protocols (Marcus A. *et al*., 2012). Briefly, 1 mL of bacterial culture, 20 hours after induction, was pelleted by centrifugation at 8000 rpm for 10 minutes. The pellet was gently resuspended in 1 mL of 15 µg/mL Congo Red (Sigma-Aldrich) solution in PBS and incubated at room temperature for 10 minutes. Subsequently, the mixture was centrifuged at 14000 rpm for 10 minutes. 150 µL of the supernatant was then transferred to a transparent 96-well plate (FALCON). The untreated Congo Red solution in PBS was used as a negative control. The absorbance of the supernatant at 490 nm was determined using a plate reader (BioTek Synergy H1 Multi-Mode Plate Reader), and the amount of bound Congo Red was quantified by subtraction.

### Whole-cell filtration ELISA

A whole-cell ELISA assay was adapted from a previously published protocol^36^ and used to quantitatively detect both the presence of extracellular assembled CsgA, and the presence of trefoil factors 1–3. PHL628-Δ*csgA* transformants were inoculated in 5 mL of LB liquid media supplemented with 100 µg/mL of carbenicillin, grown to mid-log phase and either induced with 0.3 mM of IPTG or grown without added IPTG based on optimal curli expression. Subsequently, cells were incubated at 37 °C for 20 hours before analysis. The cultures were chilled on ice and diluted to an OD_600_ of 0.3 using TBS buffer (0.05 M Tris, pH 7.4). 25 µL of the diluted samples were added to a Multiscreen-GV 96-well filter plate (0.22 µm pore size; EMD Millipore) and filtered through. The wells were washed three times with wash buffer (TBS, 0.1% Tween-20) and incubated with 200 µL of blocking solution (1% bovine serum albumin and 0.01% H_2_O_2_ in wash buffer) for 1.5 hours at 37 °C. The wells were washed three times with wash buffer, incubated with 50 µL of either anti-CsgA antibody^37^ (1:10,000 dilution in wash buffer), anti-TFF1 antibody (Sigma-Aldrich) (1:500 dilution), anti-TFF2 antibody (Sigma-Aldrich) (1:500 dilution), or anti-TFF3 antibody (Sigma-Aldrich) (1:450 dilution) for 1.5 hours at 25 °C and washed three times with wash buffer. The samples were incubated with a goat anti-rabbit HRP conjugated antibody (Thermo Fisher Scientific) (1:5,000 dilution) for 1 hour at 25 °C, washed three times with wash buffer and reacted with 100 µL of Ultra-TMB (3,3′,5,5′-tetramethylbenzidine) ELISA substrate (Thermo Fisher Scientific) at 25 °C for 20 minutes. The reaction was stopped by the addition of 50 µL of 2 M H_2_SO_4_. 100 µL of this reaction was transferred to a clear 96-well plate. Plate Reader (BioTek) was used to analyze the sample by measuring the absorbance at 450 nm and a reference wavelength at 650 nm.

### Semi-purification of curli nanofibers via filtration

The purification of curli nanofibers was adapted from a previously described method^16^. Culture expressing either the curli-TFF fusions or wild-type curli fibers was concentrated onto a 47 mm polycarbonate filter membrane with 10 μm pores (EMD Millipore) using vacuum filtration. The membrane-deposited fibers were rinsed 3 times with 25 mL of sterile DI water. Next, the filtered fibers were incubated with 5 mL of 5% (m/v) sodium dodecyl sulfate (SDS) in water for 5 min, followed by vacuum filtration of the liquid and 3 rinses with 25 mL of DI water. Semi-purified curli nanofibers were removed from the filter membrane by gently scraping the filter with a flat spatula. Purified curli nanofibers were lyophilized and stored at 4 °C.

### Adhesion of engineered bacteria to epithelial cells

10^5^ Caco-2 cells at passage 5–15 were plated in a 24-well plate (Falcon) in 500 μL of DMEM with Glutamax with 15% FBS and 1% Penicillin-Streptomycin (Gibco) and incubated at 37 °C in a 5% CO_2_ incubator for 48 hours to reach confluency. Induced bacterial cultures were centrifuged, washed with PBS, and resuspended to an OD_600_ value of 0.5 in DMEM with 1 g/L glucose and 1% FBS (Gibco). The Caco-2 monolayers were washed twice with 500 μL PBS to remove the antibiotic. 500 μL of the bacterial samples were added to the Caco-2 monolayers and incubated for 2 hours before removal by aspiration. The Caco-2 cells were washed twice with 500 μL PBS to remove unbound bacteria. In order to count the remaining viable bacteria, cells were removed from the underlying plates by exposure to 250 μL of 0.05% Trypsin-EDTA (Gibco) followed by incubation at 37 °C for 10 minutes. 750 μL of LB media were added to the well and the cells were rigorously resuspended. The samples were serially diluted and plated on antibiotic selective plates (carbenicillin, 100 μg/mL) to enumerate colony forming units (CFU) of adhered bacteria.

### Invasion of engineered bacteria into epithelial cells

The Caco-2 cell plates were prepared as described for the adhesion assay. Induced bacterial cultures were centrifuged, washed with PBS, and resuspended to an OD_600_ value of 0.5 in DMEM with 1 g/L glucose and 1% FBS (Gibco). The Caco-2 monolayers were washed twice with 500 μL PBS to remove the antibiotic. 500 μL of the bacterial samples were added to the Caco-2 monolayers and incubated for 2 hours before removal by aspiration. The Caco-2 cells were washed twice with 500 μL PBS. 500 uL of DMEM with 1 g/L glucose and 1% FBS supplemented with 100 μg/mL gentamicin (Sigma) was added to each well and incubated for 1 hour to kill extracellular bacteria. In order to count the invaded bacteria, Caco-2 cells were lysed by incubating with 1 mL of 1% Triton-X (Sigma) 37 °C for 10 minutes. The sample in each well was mixed by multiple pipetting, serially diluted and plated on antibiotic selective plates (carbenicillin, 100 μg/mL) to enumerate colony forming units (CFU) of invaded bacteria.

### Mucin binding assay

To test the adhesion of engineered curli nanofibers to mucin, bacterial cultures expressing curli fibers were transferred onto a 47 mm polycarbonate filter membranes with 10 μm pores, via vacuum filtration. The filtered biomass of engineered nanofibers was subjected to 10 mg/mL of mucin type II from porcine stomach (Sigma-Aldrich) and the mixture was co-incubated for 1.5 hours at room temperature. Mucin binding experiments were performed on wild-type curli fibers and bare untreated filter membranes to assess the degree of nonspecific binding. Next, the liquid was filtered through, and the membrane was rinsed with 3 times with 5 mL of DI water to remove non-specifically bound mucins. Finally, the membranes with engineered curli fibers and bound mucin were blocked with 5% milk in TBST overnight at 4 °C before being incubated with 5 µg/mL FITC-conjugated anti-MUC2 (MyBioSource) for 2 hours. After further washes with blocking solution, fluorescence was detected using a FluorChem M system (Protein Simple).

### Bacterial binding to gut mucosa *ex vivo*

Fresh goat colon (female, 7 months old) was obtained from local butcher (Boston, MA). After removal of fecal matter, 9 cm of colon length was excised. The colon was not washed further in order to maintain the integrity of the mucus layer. The distal end of the colon was tied off ~2 cm from the end to seal one end of the colon. 5 mL of bacteria expressing the engineered curli fibers (OD_600_ ~1 in PBS) was then added to the opening in the proximal side of colonic tube, and the proximal tube end was also tied off, leaving the fluid-filled colon with a “sausage-like” appearance. The filled “sausage” was placed gently in a 50 mL sample tube filled with PBS and subjected to light shaking for 1 hour at room temperature. Next, the “sausage” was removed from the sample tube, each end of the colon was opened, and 5 mL of PBS was flowed through the open “sausage” tube to remove unbound bacteria. The opened colon tube was then cut open longitudinally, flattened into a sheet, and placed with the mucosal side facing up in a container filled with 15 µg/mL Congo Red. After 15 minutes of incubation the tissue was rinsed thoroughly with PBS to remove unbound dye.

### Rheology of mucin-curli gels

50 mL of bacterial culture expressing curli fibers was pelleted and washed with a 50 mL of PBS. The bacterial pellet was then resuspended in 10 mL of 10 mg/mL mucin type II from porcine stomach (Sigma-Aldrich) and kept lightly shaking at room temperature for 1 hour. Next, the bacteria/mucin mixture was pelleted and the pellet was used directly for rheological measurements, performed on a TA Instruments AR-G2 rheometer with plate-plate geometry. 8 mm plates were used for strain sweeps and frequency sweeps with a gap width of 500 µm and a moisture trap. Strain sweep measurements were carried out from a strain amplitude of 0.1% to 25% at 25.0 °C (+/−0.1 °C) and a frequency of 1.0 Hz to determine small deformation linearity. Frequency sweep experiments were then performed from 0.1–100 Hz at 25.0 °C with a controlled strain amplitude of 1.0%, which was within the linear response range for all samples. All measurements were performed in triplicate.

### Cell migration assay

The human colonic carcinoma cell line Caco-2 BBE1 (ATCC) was maintained between 60–80% confluency in DMEM supplemented with 15% FBS and 1% penicillin/streptomycin (Gibco) at 37 °C in a 5% CO_2_ incubator and passaged several times before performing experiments. Next, 2 × 10^5^ cells were seeded in each well of a 6-well plate to achieve 100% confluency. Then, the medium was replaced by DMEM medium with 1% FBS and 1% penicillin/streptomycin and incubated overnight at 37 °C. After confluency was reached, a scratch (i.e. “defect”) was made in the cell monolayer with a pipette tip. After defect formation, cells were allowed to incubate for 24 hours in DMEM medium and 1% FBS along with 200 nM of semi-purified curli fibers (CsgA-TFF1, CsgA-TFF2, CsgA-TFF3, wt-CsgA) or the corresponding soluble trefoil factors (Sigma-Aldrich), respectively. Defect size after 0 and 24 hours was measured using ImageJ (NIH). Each experiment was performed in triplicate.

### Scanning electron microscopy

200 µL of curliated bacterial cultures were vacuum filtered onto Nuclepore filters (0.22 µm pore size; GE Healthcare Bio-Sciences), rinsed, and fixed with 2% formaldehyde and 2% glutaraldehyde solution overnight at 4 °C. Then, samples were washed with Millipore water for 15 minutes, dehydrated with gradient steps of ethanol (25%, 50%, 75%, 100%, 100%) – 15 minutes for each step – and dried with Critical Point Dryer (Autosamdri®-931, Tousimis®). Finally, the samples were sputter coated with 80:20 Pt:Pd and analyzed on a Zeiss Supra55VP FE-SEM.

## Electronic supplementary material

Supplemental Information

## References

[1] Johansson ME, Hansson GC (2016). Immunological aspects of intestinal mucus and mucins. Nat Rev Immunol..

[2] Caldara M (2012). Mucin biopolymers prevent bacterial aggregation by retaining cells in the free-swimming state. Curr Biol..

[3] Johansson ME (2014). Bacteria penetrate the normally impenetrable inner colon mucus layer in both murine colitis models and patients with ulcerative colitis. Gut..

[4] Marcobal A, Southwick AM, Earle KA, Sonnenburg JL (2013). A refined palate: bacterial consumption of host glycans in the gut. Glycobiology..

[5] Juge N (2012). Microbial adhesins to gastrointestinal mucus. Trends Microbiol..

[6] Pinero-Lambea C, Ruano-Gallego D, Fernandez LA (2015). Engineered bacteria as therapeutic agents. Curr Opin Biotechnol..

[7] Turroni F (2013). Role of sortase-dependent pili of Bifidobacterium bifidum PRL2010 in modulating bacterium-host interactions. Proc Natl Acad Sci USA.

[8] Tukel C (2010). Toll-like receptors 1 and 2 cooperatively mediate immune responses to curli, a common amyloid from enterobacterial biofilms. Cell Microbiol..

[9] Claesen J, Fischbach MA (2015). Synthetic microbes as drug delivery systems. ACS Synth Biol..

[10] Maxmen A (2017). Living therapeutics: Scientists genetically modify bacteria to deliver drugs. Nat Med..

[11] Duan FF, Liu JH, March JC (2015). Engineered commensal bacteria reprogram intestinal cells into glucose-responsive insulin-secreting cells for the treatment of diabetes. Diabetes..

[12] Braat H (2006). A phase I trial with transgenic bacteria expressing interleukin-10 in Crohn’s disease. Clin Gastroenterol Hepatol..

[13] Ou B (2016). Genetic engineering of probiotic Escherichia coli Nissle 1917 for clinical application. Appl Microbiol Biotechnol..

[14] Barnhart MM, Chapman MR (2006). Curli biogenesis and function. Annu Rev Microbiol..

[15] Nguyen PQ, Botyanszki Z, Tay PK, Joshi NS (2014). Programmable biofilm-based materials from engineered curli nanofibres. Nat Commun..

[16] Courchesne, N.-M. D. *et al*. Scalable Production of Genetically Engineered Nanofibrous Macroscopic Materials viaFiltration. *ACS Biomater. Sci. Eng*. 3, 733–741 (2017). 10.1021/acsbiomaterials.6b00437 33440494

[17] Tomasetto C (2000). pS2/TFF1 interacts directly with the VWFC cysteine-rich domains of mucins. Gastroenterology..

[18] Newton JL, Allen A, Westley BR, May FE (2000). The human trefoil peptide, TFF1, is present in different molecular forms that are intimately associated with mucus in normal stomach. Gut..

[19] Kindon H, Pothoulakis C, Thim L, Lynch-Devaney K, Podolsky DK (1995). Trefoil peptide protection of intestinal epithelial barrier function: cooperative interaction with mucin glycoprotein. Gastroenterology..

[20] Thim L, Madsen F, Poulsen SS (2002). Effect of trefoil factors on the viscoelastic properties of mucus gels. Eur J Clin Invest..

[21] Hoffmann W (2005). Trefoil factors TFF (trefoil factor family) peptide-triggered signals promoting mucosal restitution. Cell Mol Life Sci..

[22] Meyer zum Buschenfelde D, Tauber R, Huber O (2006). TFF3-peptide increases transepithelial resistance in epithelial cells by modulating claudin-1 and -2 expression. Peptides..

[23] Aamann L, Vestergaard EM, Gronbaek H (2014). Trefoil factors in inflammatory bowel disease. World J Gastroenterol..

[24] Conway, T. & Cohen, P. S. Commensal and Pathogenic Escherichia coli Metabolism in theGut. *Microbiol Spectr*. **3** (2015). 10.1128/microbiolspec.MBP-0006-2014 PMC4510460 26185077

[25] Vidal O (1998). Isolation of an Escherichia coli K-12 mutant strain able to form biofilms on inert surfaces: involvement of a new ompR allele that increases curli expression. J Bacteriol..

[26] Li H (2015). The outer mucus layer hosts a distinct intestinal microbial niche. Nat Commun..

[27] Kjellev S, Nexo E, Thim L, Poulsen SS (2006). Systemically administered trefoil factors are secreted into the gastric lumen and increase the viscosity of gastric contents. Br J Pharmacol..

[28] Kline KA, Falker S, Dahlberg S, Normark S, Henriques-Normark B (2009). Bacterial adhesins in host-microbe interactions. Cell Host Microbe..

[29] Oertel M (2001). Trefoil factor family-peptides promote migration of human bronchial epithelial cells: synergistic effect with epidermal growth factor. Am J Respir Cell Mol Biol..

[30] Yu G (2010). Cell migration-promoting and apoptosis-inhibiting activities of Bm-TFF2 require distinct structure basis. Biochem Biophys Res Commun..

[31] Hoffmann W (2009). Trefoil factor family (TFF) peptides and chemokine receptors: a promising relationship. J Med Chem..

[32] Taupin D, Podolsky DK (2003). Trefoil factors: initiators of mucosal healing. Nat Rev Mol Cell Biol..

[33] Van Gerven N (2014). Secretion and functional display of fusion proteins through the curli biogenesis pathway. Molecular Microbiology..

[34] Botyanszki Z, Tay PK, Nguyen PQ, Nussbaumer MG, Joshi NS (2015). Engineered catalytic biofilms: Site-specific enzyme immobilization onto *E. coli* curli nanofibers. Biotechnol Bioeng..

[35] Gibson DG (2009). Enzymatic assembly of DNA molecules up to several hundred kilobases. Nat Methods..

[36] Itoh S (2002). New rapid enzyme-linked immunosorbent assay to detect antibodies against bacterial surface antigens using filtration plates. Biol Pharm Bull..

[37] Chapman MR (2002). Role of Escherichia coli curli operons in directing amyloid fiber formation. Science..

