# Author Correction: Modulating bacterial and gut mucosal interactions with engineered biofilm matrix proteins

**DOI:** 10.1038/s41598-021-96036-w

**Published:** 2021-09-06

**Authors:** Anna M. Duraj-Thatte, Pichet Praveschotinunt, Trevor R. Nash, Frederick R. Ward, Peter Q. Nguyen, Neel S. Joshi

**Affiliations:** 1 Wyss Institute for Biologically Inspired Engineering, Harvard University, Boston, MA USA; 2 John A. Paulson School of Engineering and Applied Sciences, Harvard University, Cambridge, MA USA

Correction to: *Scientific Reports* 10.1038/s41598-018-21834-8, published on 22 February 2018

Peter Q. Nguyen was omitted from the author list in the original version of this Article.

The Author Contribution section now reads:

“A.D.-T. and N.S.J. conceived the idea and designed the experiments. A.D.-T., and P.P. conducted all experiments and data analysis. T.N. and F.W. cloned the TFF variants. P.Q.N. designed and provided template for the flexible linker. The manuscript has been approved by all the authors.”

The original Article has been corrected.

