# Supplementary material for: Modulating bacterial and gut mucosal interactions with engineered biofilm matrix proteins
Source: Sci Rep. 2018 Feb 22;8:3475. doi: 10.1038/s41598-018-21834-8 (PMC5823925; doi:10.1038/s41598-018-21834-8)
Supplement: Supplementary file 1 — Supplemental Information [file 41598_2018_21834_MOESM1_ESM.pdf]

Supplemental Information

Modulating bacterial and gut mucosal interactions with engineered biofilm matrix proteins

Anna Duraj-Thatte[1,2], Pichet Praveschotinunt [1,2], Trevor R. Nash[1,2], Frederick R. Ward[2], Peter Q. Nguyen[1,2], Neel S. Joshi[1,2]

[1] Wyss Institute for Biologically Inspired Engineering, Harvard University, Boston, MA, United States;

[2] School of Engineering and Applied Sciences, Harvard University, Cambridge, MA, United States

Supplementary figure 1 pBbE1a plasmid maps containing wild-type CsgA and CsgA variants in this study

A

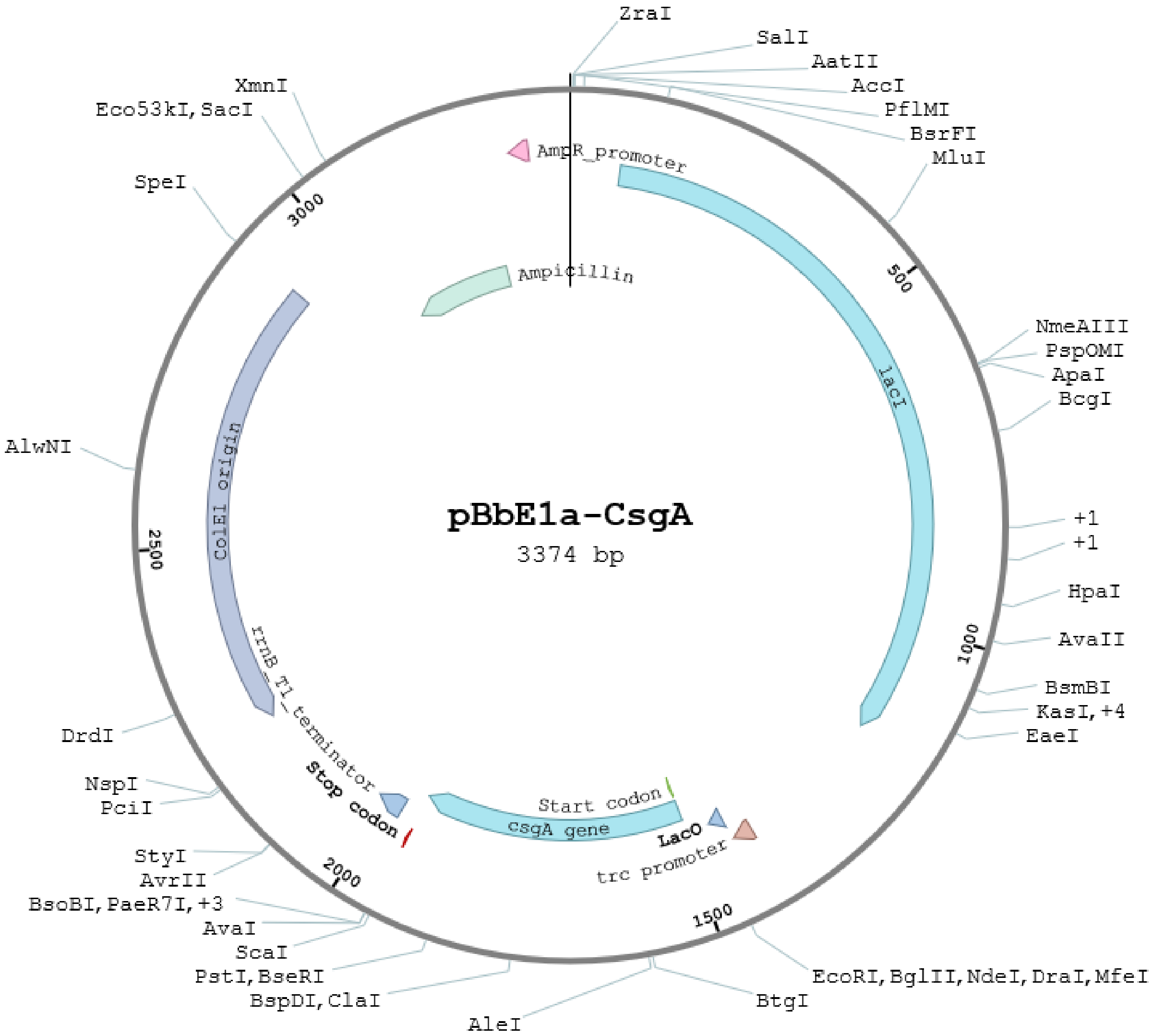

B

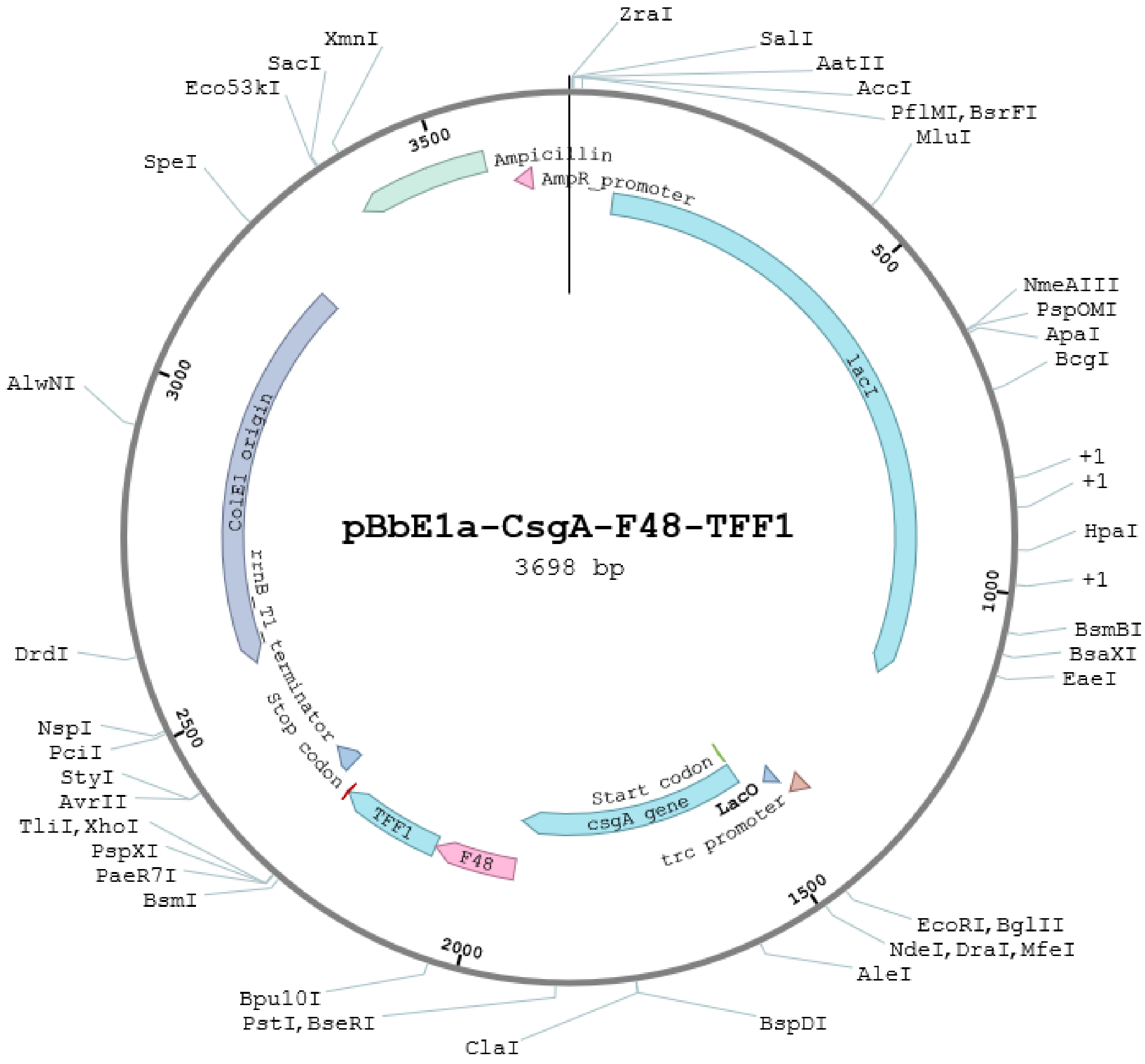
pBbE1a-CsgA-F48-TFF1
3698 bp
ZraI
SalI
AatII
AccI
PflMI, BsrFI
MluI
NmeAIII
PspOMI
ApaI
BcgI
+1
+1
HpaI
+1
BsmBI
BsaXI
EaeI
EcoRI, BglII
NdeI, DraI, MfeI
AleI
BspDI
ClaI
PstI, BseRI
Bpu10I
BsmI
PaeR7I
PspXI
TliI, XhoI
AvrII
StyI
PciI
NspI
DrdI
AlwNI
SpeI
Eco53kI
SacI
XmnI
500
1000
1500
2000
2500
3000
3500
Ampicillin
AmpR_promoter
lacI
Start codon
csgA gene
LacO
trc promoter
F48
TFF1
Stop codon
rrnB_T1_terminator
ColE1 origin

C

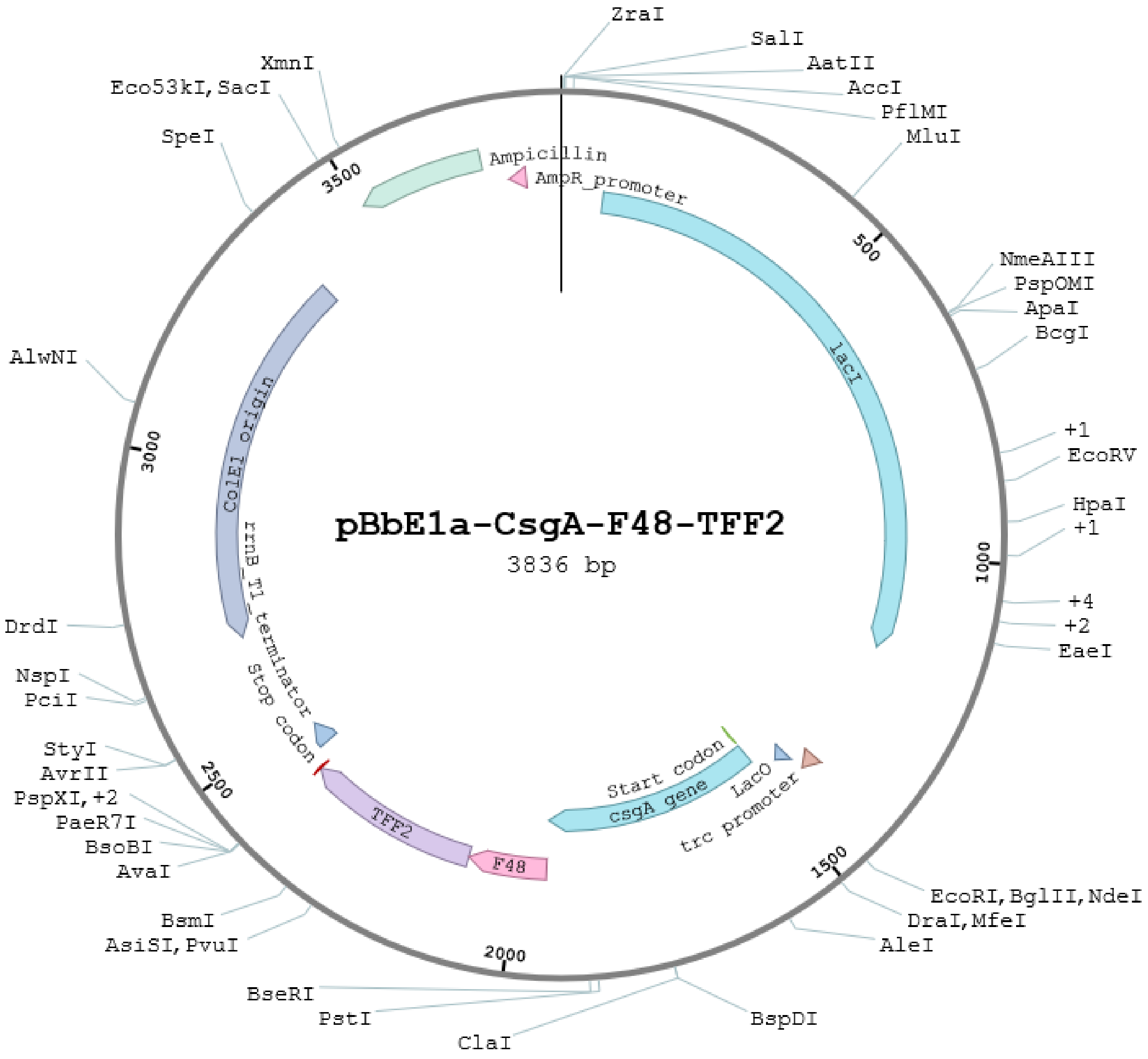
pBbE1a-CsgA-F48-TFF2
3836 bp
ZraI
SalI
AatII
AccI
PflMI
MluI
NmeAIII
PspOMI
ApaI
BcgI
+1
EcoRV
HpaI
+1
+4
+2
EaeI
EcoRI, BglII, NdeI
DraI, MfeI
AleI
BspDI
ClaI
PstI
BseRI
AsiSI, PvuI
BsmI
AvaI
BsoBI
PaeR7I
PspXI, +2
AvrII
StyI
PciI
NspI
DrdI
AlwNI
SpeI
Eco53kI, SacI
XmnI
Ampicillin
AmpR_promoter
lacI
Start codon
csgA gene
LacO
trc promoter
F48
TFF2
Stop codon
rrnB_T1_terminator
ColE1 origin
500
1000
1500
2000
2500
3000
3500

D

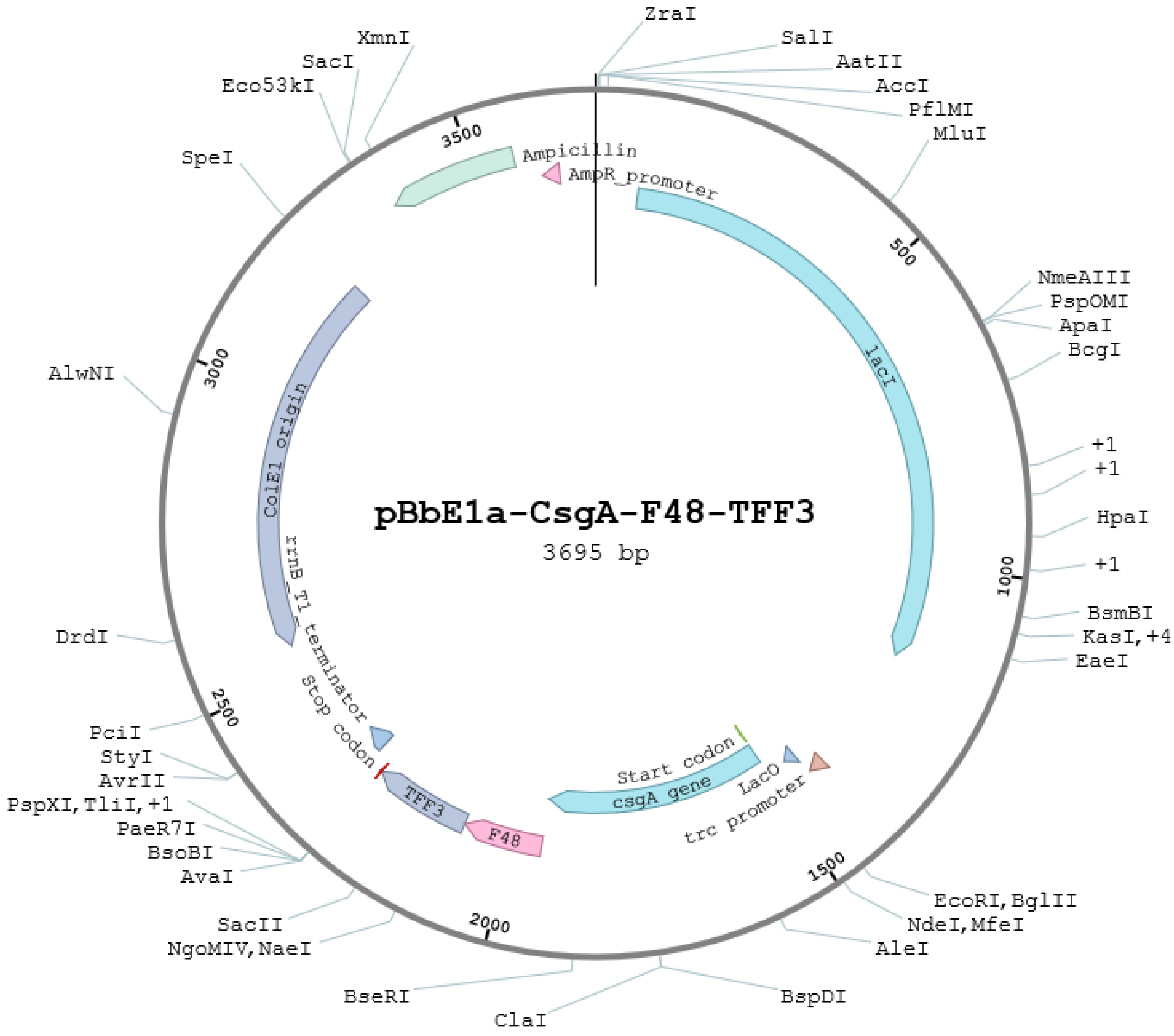
pBbE1a-CsgA-F48-TFF3
3695 bp
ZraI
SalI
AatII
AccI
PflMI
MluI
NmeAIII
PspOMI
ApaI
BcgI
+1
+1
HpaI
+1
BsmBI
KasI,+4
EaeI
EcoRI,BglII
NdeI,MfeI
AleI
BspDI
ClaI
BseRI
NgoMIV,NaeI
SacII
AvaI
BsoBI
PaeR7I
PspXI,TliI,+1
AvrII
StyI
PciI
DrdI
AlwNI
SpeI
Eco53kI
SacI
XmnI
Ampicillin
AmpR_promoter
lacI
ColE1 origin
rrnB_T1_terminator
Stop codon
TFF3
F48
csgA gene
Start codon
LacO
trc promoter
500
1000
1500
2000
2500
3000
3500

Supplementary figure 1 (A) Plasmid map of pBbE1a vector containing wild type CsgA, (B) Plasmid map of pBbE1a vector containing CsgA fused with flexible linker (48 aa) and trefoil factor-1 at C-terminus, (C) Plasmid map of pBbE1a vector containing CsgA fused with flexible linker (48 aa) and trefoil factor-2 at C-terminus, (D) Plasmid map of pBbE1a vector containing CsgA fused with flexible linker (48 aa) and trefoil factor-3 at C-terminus

Supplementary figure 2 Growth curve of PHL628 Δ*csgA* variants with and without IPTG induction

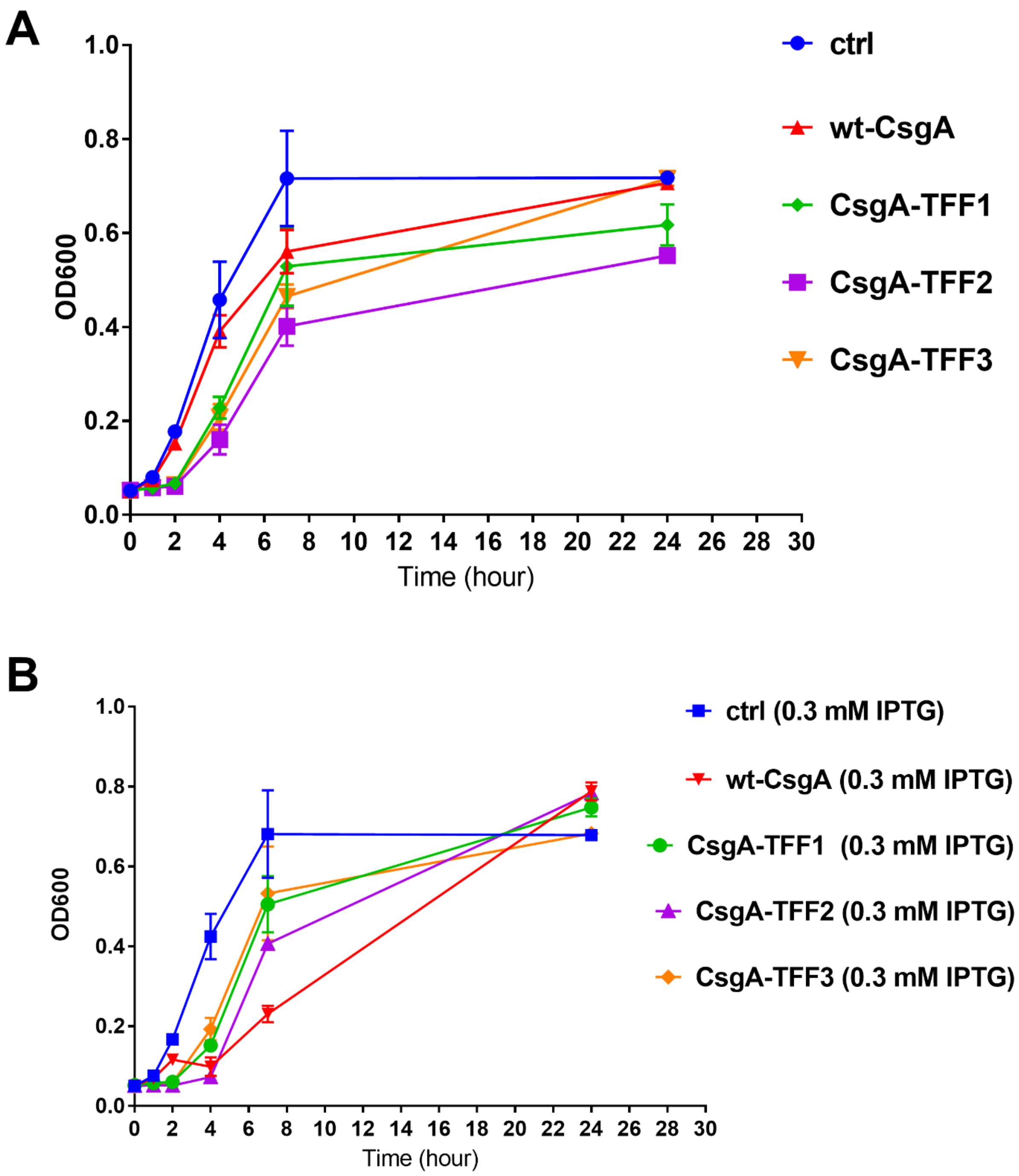

Supplementary figure 2 (A) the growth curve indicating OD600 of PHL628 Δ*csgA* variants over time in Luria broth at 37°C 225 rpm shaking incubator, (B) the growth curve indicating OD600 of PHL628 Δ*csgA* variants over time in Luria broth with 0.3 mM Isopropyl β-D-1-thiogalactopyranoside (IPTG) at 37°C 225 rpm shaking incubator

Supplementary figure 3. Cell migration assay with soluble trefoil factors.

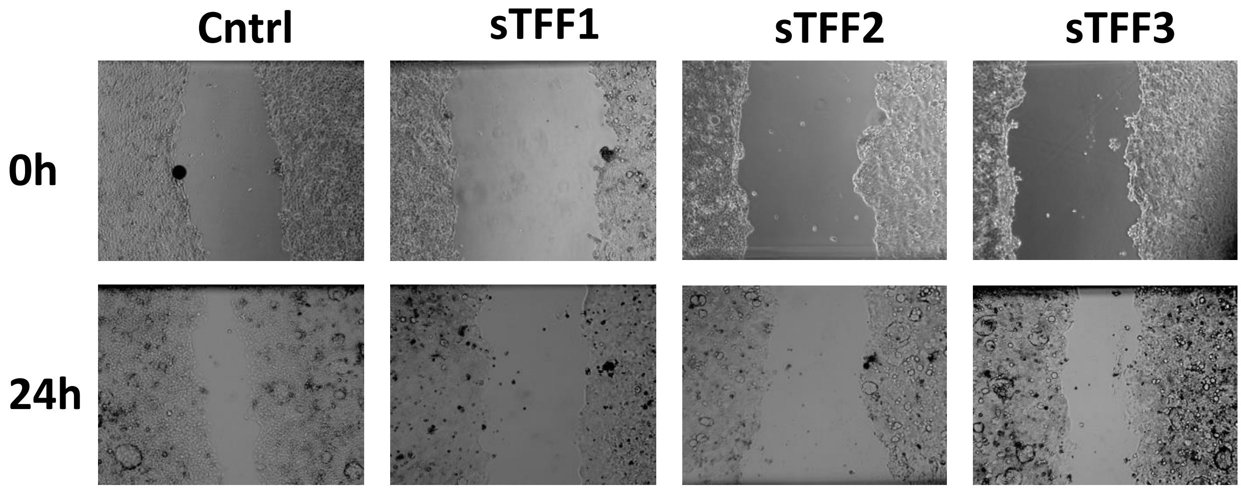

Supplementary table 1 Amino acid sequence of wild-type CsgA and CsgA variants in this study

CsgA Variants	Amino acid sequence
Wild-type CsgA	MKLLKVAAIAAIVFSGSALAGVVPQYGGGGNHGGGGNNSGPNSE<br>LNIYQYGGGNSALALQTDARNSDLTITQHGGGNGADVGQGSDDS<br>SIDLTQRGFGNSATLDQWNGKNSEMTVKQFGGGNGAAVDQTAS<br>NSSVNVTQVGFGNNATAHQY
CsgA-F48-TFF1	MKLLKVAAIAAIVFSGSALAGVVPQYGGGGNHGGGGNNSGPNSE<br>LNIYQYGGGNSALALQTDARNSDLTITQHGGGNGADVGQGSDDS<br>SIDLTQRGFGNSATLDQWNGKNSEMTVKQFGGGNGAAVDQTAS<br>NSSVNVTQVGFGNNATAHQYGGGSGGGSGGGSGGGSGGGSGGG<br>SGGGSGGGSGGGSGGGSGGGSGGGSEAQTETCTVAPRERQNCGF<br>PGVTPSQCANKGCCFDDTVRGVPWCFYPNTIDVPPEEECEF
CsgA-F48-TFF2	MKLLKVAAIAAIVFSGSALAGVVPQYGGGGNHGGGGNNSGPNSE<br>LNIYQYGGGNSALALQTDARNSDLTITQHGGGNGADVGQGSDDS<br>SIDLTQRGFGNSATLDQWNGKNSEMTVKQFGGGNGAAVDQTAS<br>NSSVNVTQVGFGNNATAHQYGGGSGGGSGGGSGGGSGGGSGGG<br>SGGGSGGGSGGGSGGGSGGGSGGGSEKPSPCQCSRLSPHNRTNCG<br>FPGITSDQCFDNGCCFDSSVTGVPWCFHPLPKQESDQCVMEVSDR<br>RNCGYPGISPEECASRKCCFSNFIFEVPWCFFPKSVEDCHY
CsgA-F48-TFF3	MKLLKVAAIAAIVFSGSALAGVVPQYGGGGNHGGGGNNSGPNSE<br>LNIYQYGGGNSALALQTDARNSDLTITQHGGGNGADVGQGSDDS<br>SIDLTQRGFGNSATLDQWNGKNSEMTVKQFGGGNGAAVDQTAS<br>NSSVNVTQVGFGNNATAHQYGGGSGGGSGGGSGGGSGGGSGGG<br>SGGGSGGGSGGGSGGGSGGGSGGGSEEYVGLSANQCAVPAKDRV<br>DCGYPHVTPKECNNRGCCFDSRIPGVPWCFKPLQEAECTF

Supplementary information 1 DNA sequence of pBbE1a plasmids containing wild-type CsgA and CsgA variants in this study

>pBbE1a-CsgA

gacgtcgacaccatcgaatggtgcaaaacctttcgcggtatggcatgatagcgcccggaagagagtcaattcagggtgg

tgaatgtgaaaccagtaacgttatacgatgtcgcagagtatgccggtgtctcttatcagaccgtttcccgcgtggtgaa

ccaggccagccacgtttctgcgaaaacgcgggaaaaagtggaagcggcgatggcggagctgaattacattcccaaccgc

gtggcacaacaactggcgggcaaacagtcgttgctgattggcgttgccacctccagtctggccctgcacgcgccgtcgc

aaattgtcgcggcgattaaatctcgcgccgatcaactgggtgccagcgtggtggtgtcgatggtagaacgaagcggcgt

cgaagcctgtaaagcggcggtgcacaatcttctcgcgcaacgcgtcagtgggctgatcattaactatccgctggatgac

caggatgccattgctgtggaagctgcctgcactaatgttccggcgttatttcttgatgtctctgaccagacacccatca

acagtattattttctcccatgaagacggtacgcgactgggcgtggagcatctggtcgcattgggtcaccagcaaatcgc

gctgttagcgggcccattaagttctgtctcggcgcgtctgcgtctggctggctggcataaatatctcactcgcaatcaa

attcagccgatagcggaacgggaaggcgactggagtgccatgtccggttttcaacaaaccatgcaaatgctgaatgagg

gcatcgttcccactgcgatgctggttgccaacgatcagatggcgctgggcgcaatgcgcgccattaccgagtccgggct

gcgcgttggtgcggatatctcggtagtgggatacgacgataccgaagacagctcatgttatatcccgccgttaaccacc

atcaaacaggattttcgcctgctggggcaaaccagcgtggaccgcttgctgcaactctctcagggccaggcggtgaagg

gcaatcagctgttgcccgtctcactggtgaaaagaaaaaccaccctggcgcccaatacgcaaaccgcctctccccgcgc

gttggccgattcattaatgcagctggcacgacaggtttcccgactggaaagcgggcagtgagcgcaacgcaattaatgt

aagttagcgcgaattgatctggtttgacagcttatcatcgactgcacggtgcaccaatgcttctggcgtcaggcagcca

tcggaagctgtggtatggctgtgcaggtcgtaaatcactgcataattcgtgtcgctcaaggcgcactcccgttctggat

aatgtttttgcgccgacatcataacggttctggcaaatattctgaaatgagctgttgacaattaatcatccggctcgt

ataatgtgtggaattgtgagcggataacaatttcagaattcaaaagatcttttaagaaggagatatacatATGAAACTT

TTAAAAGTAGCAGCAATTGCAGCAATCGTATTCTCCGGTAGCGCTCTGGCAGGTGTT GTTCCTCAGTACGGCGGCGGCG

GTAACCACGGTGGTGGCGGTAATAATAGCGGCCCAAATTCTGAGCTGAACATTTACC AGTACGGTGGCGGTAACTCTGC

ACTTGCTCTGCAAACTGATGCCCGTAACTCTGACTTGACTATTACCCAGCATGGCGG CGGTAATGGTGCAGATGTTGGT

CAGGGCTCAGATGACAGCTCAATCGATCTGACCCAACGTGGCTTCGGTAACAGCGC TACTCTTGATCAGTGGAACGGCA

AAAATTCTGAAATGACGGTTAAACAGTTCGGTGGTGGCAACGGTGCTGCAGTTGAC CAGACTGCATCTAACTCCTCCGT

CAACGTGACTCAGGTTGGCTTTGGTAACAACGCGACCGCTCATCAGTACTGACTCGA Gtaaggatctccaggcatcaaa

taaaacgaaaggctcagtcgaaagactgggcctttcgttttatctgttgtttgtcggtgaacgctctctactagagtca

cactggctcaccttcgggtgggcctttctgcgtttatacctagggcgttcggctgcggcgagcggtatcagctcactca

aaggcggtaatacggttatccacagaatcaggggataacgcaggaaagaacatgtgagcaaaaggccagcaaaaggcca

ggaaccgtaaaaaggccgcgttgctggcgtttttccataggctccgcccccctgacgagcatcacaaaaatcgacgctc

aagtcagaggtggcgaaacccgacaggactataaagataccaggcgtttccccctggaagctccctcgtgcgctctcct

gttccgaccctgccgcttaccggatacctgtccgcctttctcccttcgggaagcgtggcgctttctcatagctcacgct

gtaggtatctcagttcggtgtaggtcgttcgctccaagctgggctgtgtgcacgaaccccccgttcagcccgaccgctg

cgccttatccggtaactatcgtcttgagtccaacccggtaagacacgacttatcgccactggcagcagccactggtaac

aggattagcagagcgaggtatgtaggcggtgctacagagttcttgaagtggtggcctaactacggctacactagaagga

cagtatttggtatctgcgctctgctgaagccagttaccttcggaaaaagagttggtagctcttgatccggcaaacaaac

caccgctggtagcggtggtttttttgtttgcaagcagcagattacgcgcagaaaaaaaggatctcaagaagatcctttg

atcttttctacggggtctgacgctcagtggaacgaaaactcacgttaagggattttggtcatgactagtgcttggattc

tcaccaataaaaaacgcccggcggcaaccgagcgttctgaacaaatccagatggagttctgaggtcattactggatcta

tcaacaggagtccaagcgagctcgtaaacttggtctgacagttacattggaaaacgttcttcggggcgaaaactctcaa

ggatcttaccgctgttgagatccagttcgatgtaacccactcgtgcacccaactgatcttcagcatcttttactttcac

cagcgtttctgggtgagcaaaaacaggaaggcaaaatgccgcaaaaaagggaataagggcgacacggaaatgttgaata

ctcatactcttcctttttcaatattattgaagcatttatcagggttattgtctcatgagcggatacatatttgaatgta

tttagaaaaataaacaaataggggttccgcgcacatttccccgaaaagtgccacct

>pBbE1a-CsgA-F48-TFF1

gacgtcgacaccatcgaatggtgcaaaacctttcgcggtatggcatgatagcgcccggaagagagtcaattcagggtgg

tgaatgtgaaaccagtaacgttatacgatgtcgcagagtatgccggtgtctcttatcagaccgtttcccgcgtggtgaa

ccaggccagccacgtttctgcgaaaacgcgggaaaaagtggaagcggcgatggcggagctgaattacattcccaaccgc

gtggcacaacaactggcgggcaaacagtcgttgctgattggcgttgccacctccagtctggccctgcacgcgccgtcgc

aaattgtcgcggcgattaaatctcgcgccgatcaactgggtgccagcgtggtggtgtcgatggtagaacgaagcggcgt

cgaagcctgtaaagcggcggtgcacaatcttctcgcgcaacgcgtcagtgggctgatcattaactatccgctggatgac

caggatgccattgctgtggaagctgcctgcactaatgttccggcgttatttcttgatgtctctgaccagacacccatca

acagtattattttctcccatgaagacggtacgcgactgggcgtggagcatctggtcgcattgggtcaccagcaaatcgc

gctgttagcgggcccattaagttctgtctcggcgcgtctgcgtctggctggctggcataaatatctcactcgcaatcaa

attcagccgatagcggaacgggaaggcgactggagtgccatgtccggttttcaacaaaccatgcaaatgctgaatgagg

gcatcgttcccactgcgatgctggttgccaacgatcagatggcgctgggcgcaatgcgcgccattaccgagtccgggct

gcgcgttggtgcggatatctcggtagtgggatacgacgataccgaagacagctcatgttatatcccgccgttaaccacc

atcaaacaggattttcgcctgctggggcaaaccagcgtggaccgcttgctgcaactctctcagggccaggcggtgaagg

gcaatcagctgttgcccgtctcactggtgaaaagaaaaaccaccctggcgcccaatacgcaaaccgcctctccccgcgc

gttggccgattcattaatgcagctggcacgacaggtttcccgactggaaagcgggcagtgagcgcaacgcaattaatgt

aagttagcgcgaattgatctggtttgacagcttatcatcgactgcacggtgcaccaatgcttctggcgtcaggcagcca

tcggaagctgtggtatggctgtgcaggtcgtaaatcactgcataattcgtgtcgctcaaggcgcactcccgttctggat

aatgttttttgcgccgacatcataacggttctggcaaatattctgaaatgagctgttgacaattaatcatccggctcgt

ataatgtgtggaattgtgagcggataacaatttcagaattcaaaagatcttttaagaaggagatatacatATGAAACTT

TTAAAAGTAGCAGCAATTGCAGCAATCGTATTCTCCGGTAGCGCTCTGGCAGGTGTT
GTTCCTCAGTACGGCGGCGGCG

GTAACCACGGTGGTGGCGGTAATAATAGCGGCCCAAATTCTGAGCTGAACATTTACC
AGTACGGTGGCGGTAACTCTGC

ACTTGCTCTGCAAACTGATGCCCGTAACTCTGACTTGACTATTACCCAGCATGGCGG
CGGTAATGGTGCAGATGTTGGT

CAGGGCTCAGATGACAGCTCAATCGATCTGACCCAACGTGGCTTCGGTAACAGCGC
TACTCTTGATCAGTGGAACGGCA

AAAATTCTGAAATGACGGTTAAACAGTTCGGTGGTGGCAACGGTGCTGCAGTTGAC
CAGACTGCATCTAACTCCTCCGT

CAACGTGACTCAGGTTGGCTTTGGTAACAACGCGACCGCTCATCAGTACGGTGGTGG
TAGTGGTGGCGGCAGTGGTGGC

GGTAGCGGCGGTGGCTCCGGTGGCGGTTCTGGCGGCGGTTCTGGTGGTGGTTCTGGC
GGTGGCTCAGGTGGGGGTTCCG

GCGGCGGTAGCGGCGGTGGATCTGGCGGCGGCTCTGAAGCGCAGACCGAAACCTGC
ACCGTGGCGCCGCGCGAACGCCA

GAACTGCGGCTTTCCGGGCGTGACCCCGAGCCAGTGCGCGAACAAAGGCTGCTGCT
TTGATGATACCGTGCGCGGCGTG

CCGTGGTGCTTTTATCCGAACACCATTGATGTGCCGCCGGAAGAAGAATGCGAATTT
TGACTCGAGtaaggatctccag

gcatcaaataaaacgaaaggctcagtcgaaagactgggcctttcgttttatctgttgtttgtcggtgaacgctctctac

tagagtcacactggctcaccttcgggtgggcctttctgcgtttatacctagggcgttcggctgcggcgagcggtatcag

ctcactcaaaggcggtaatacggttatccacagaatcaggggataacgcaggaaagaacatgtgagcaaaaggccagca

aaaggccaggaaccgtaaaaaggccgcgttgctggcgtttttccataggctccgcccccctgacgagcatcacaaaaat

cgacgctcaagtcagaggtggcgaaacccgacaggactataaagataccaggcgtttccccctggaagctccctcgtgc

gctctcctgttccgaccctgccgcttaccggatacctgtccgcctttctcccttcgggaagcgtggcgctttctcatag

ctcacgctgtaggtatctcagttcggtgtaggtcgttcgctccaagctgggctgtgtgcacgaaccccccgttcagccc

gaccgctgcgccttatccggtaactatcgtcttgagtccaacccggtaagacacgacttatcgccactggcagcagcca

ctggtaacaggattagcagagcgaggtatgtaggcggtgctacagagttcttgaagtggtggcctaactacggctacac

tagaaggacagtatttggtatctgcgctctgctgaagccagttaccttcggaaaaagagttggtagctcttgatccggc

aaacaaaccaccgctggtagcggtggtttttttgtttgcaagcagcagattacgcgcagaaaaaaaggatctcaagaag

atcctttgatcttttctacggggtctgacgctcagtggaacgaaaactcacgttaagggattttggtcatgactagtgc

ttggattctcaccaataaaaaacgcccggcggcaaccgagcgttctgaacaaatccagatggagttctgaggtcattac

tggatctatcaacaggagtccaagcgagctcgtaaacttggtctgacagttacattggaaaacgttcttcggggcgaaa

actctcaaggatcttaccgctgttgagatccagttcgatgtaacccactcgtgcacccaactgatcttcagcatctttt

actttcaccagcgtttctgggtgagcaaaaacaggaaggcaaaatgccgcaaaaaagggaataagggcgacacggaaat

gttgaatactcatactcttcctttttcaatattattgaagcatttatcagggttattgtctcatgagcggatacatatt

tgaatgtatttagaaaaataaacaaataggggttccgcgcacatttccccgaaaagtgccacct

>pBbE1a-CsgA-F48-TFF2

gacgtcgacaccatcgaatggtgcaaaacctttcgcggtatggcatgatagcgcccggaagagagtcaattcagggtgg

tgaatgtgaaaccagtaacgttatacgatgtcgcagagtatgccggtgtctcttatcagaccgtttcccgcgtggtgaa

ccaggccagccacgtttctgcgaaaacgcgggaaaaagtggaagcggcgatggcggagctgaattacattcccaaccgc

gtggcacaacaactggcgggcaaacagtcgttgctgattggcgttgccacctccagtctggccctgcacgcgccgtcgc

aaattgtcgcggcgattaaatctcgcgccgatcaactgggtgccagcgtggtggtgtcgatggtagaacgaagcggcgt

cgaagcctgtaaagcggcggtgcacaatcttctcgcgcaacgcgtcagtgggctgatcattaactatccgctggatgac

caggatgccattgctgtggaagctgcctgcactaatgttccggcgttatttcttgatgtctctgaccagacacccatca

acagtattattttctcccatgaagacggtacgcgactgggcgtggagcatctggtcgcattgggtcaccagcaaatcgc

gctgttagcgggcccattaagttctgtctcggcgcgtctgcgtctggctggctggcataaatatctcactcgcaatcaa

attcagccgatagcggaacgggaaggcgactggagtgccatgtccggttttcaacaaaccatgcaaatgctgaatgagg

gcatcgttcccactgcgatgctggttgccaacgatcagatggcgctgggcgcaatgcgcgccattaccgagtccgggct

gcgcgttggtgcggatatctcggtagtgggatacgacgataccgaagacagctcatgttatatcccgccgttaaccacc

atcaaacaggattttcgcctgctggggcaaaccagcgtggaccgcttgctgcaactctctcagggccaggcggtgaagg

gcaatcagctgttgcccgtctcactggtgaaaagaaaaaccaccctggcgcccaatacgcaaaccgcctctccccgcgc

gttggccgattcattaatgcagctggcacgacaggtttcccgactggaaagcgggcagtgagcgcaacgcaattaatgt

aagttagcgcgaattgatctggtttgacagcttatcatcgactgcacggtgcaccaatgcttctggcgtcaggcagcca

tcggaagctgtggtatggctgtgcaggtcgtaaatcactgcataattcgtgtcgctcaaggcgcactcccgttctggat

aatgttttttgcgccgacatcataacggttctggcaaatattctgaaatgagctgttgacaattaatcatccggctcgt

ataatgtgtggaattgtgagcggataacaatttcagaattcaaaagatcttttaagaaggagatatacatATGAAACTT

TTAAAAGTAGCAGCAATTGCAGCAATCGTATTCTCCGGTAGCGCTCTGGCAGGTGTT
GTTCCTCAGTACGGCGGCGGCG

GTAACCACGGTGGTGGCGGTAATAATAGCGGCCCAAATTCTGAGCTGAACATTTACC
AGTACGGTGGCGGTAACTCTGC

ACTTGCTCTGCAAACTGATGCCCGTAACTCTGACTTGACTATTACCCAGCATGGCGG
CGGTAATGGTGCAGATGTTGGT

CAGGGCTCAGATGACAGCTCAATCGATCTGACCCAACGTGGCTTCGGTAACAGCGC
TACTCTTGATCAGTGGAACGGCA

AAAATTCTGAAATGACGGTTAAACAGTTCGGTGGTGGCAACGGTGCTGCAGTTGAC
CAGACTGCATCTAACTCCTCCGT

CAACGTGACTCAGGTTGGCTTTGGTAACAACGCGACCGCTCATCAGTACGGTGGTGG
TAGTGGTGGCGGCAGTGGTGGC

GGTAGCGGCGGTGGCTCCGGTGGCGGTTCTGGCGGCGGTTCTGGTGGTGGTTCTGGC
GGTGGCTCAGGTGGGGGTTCCG

GCGGCGGTAGCGGCGGTGGATCTGGCGGCGGCTCTGAAAAACCGAGCCCGTGCCAG
TGCAGCCGCCTGAGCCCGCATAA

CCGCACCAACTGCGGCTTTCCGGGCATTACCAGCGATCAGTGCTTTGATAACGGCTG
CTGCTTTGATAGCAGCGTGACC

GGCGTGCCGTGGTGCTTTCATCCGCTGCCGAAACAGGAAAGCGATCAGTGCGTGAT
GGAAGTGAGCGATCGCCGCAACT

GCGGCTATCCGGGCATTAGCCCGGAAGAATGCGCGAGCCGCAAATGCTGCTTTAGC
AACTTTATTTTTGAAGTGCCGTG

GTGCTTTTTTCCGAAAAGCGTGGAAGATTGCCATTATTGACTCGAGtaaggatctccaggcatc
aaataaaacgaaagg

ctcagtcgaaagactgggcctttcgttttatctgttgtttgtcggtgaacgctctctactagagtcacactggctcacc

ttcgggtgggcctttctgcgtttatacctagggcgttcggctgcggcgagcggtatcagctcactcaaaggcggtaata

cggttatccacagaatcaggggataacgcaggaaagaacatgtgagcaaaaggccagcaaaaggccaggaaccgtaaaa

aggccgcgttgctggcgtttttccataggctccgcccccctgacgagcatcacaaaaatcgacgctcaagtcagaggtg

gcgaaacccgacaggactataaagataccaggcgtttccccctggaagctccctcgtgcgctctcctgttccgaccctg

ccgcttaccggatacctgtccgcctttctcccttcgggaagcgtggcgctttctcatagctcacgctgtaggtatctca

gttcggtgtaggtcgttcgctccaagctgggctgtgtgcacgaaccccccgttcagcccgaccgctgcgccttatccgg

taactatcgtcttgagtccaacccggtaagacacgacttatcgccactggcagcagccactggtaacaggattagcaga

gcgaggtatgtaggcggtgctacagagttcttgaagtggtggcctaactacggctacactagaaggacagtatttggta

tctgcgctctgctgaagccagttaccttcggaaaaagagttggtagctcttgatccggcaaacaaaccaccgctggtag

cggtggtttttttgtttgcaagcagcagattacgcgcagaaaaaaaggatctcaagaagatcctttgatcttttctacg

gggtctgacgctcagtggaacgaaaactcacgttaagggattttggtcatgactagtgcttggattctcaccaataaaa

aacgcccggcggcaaccgagcgttctgaacaaatccagatggagttctgaggtcattactggatctatcaacaggagtc

caagcgagctcgtaaacttggtctgacagttacattggaaaacgttcttcggggcgaaaactctcaaggatcttaccgc

tgttgagatccagttcgatgtaacccactcgtgcacccaactgatcttcagcatcttttactttcaccagcgtttctgg

gtgagcaaaaacaggaaggcaaaatgccgcaaaaaagggaataagggcgacacggaaatgttgaatactcatactcttc

ctttttcaatattattgaagcatttatcagggttattgtctcatgagcggatacatatttgaatgtatttagaaaaata

aacaaataggggttccgcgcacatttccccgaaaagtgccacct

>pBbE1a-CsgA-F48-TFF3

gacgtcgacaccatcgaatggtgcaaaacctttcgcggtatggcatgatagcgcccggaagagagtcaattcagggtgg

tgaatgtgaaaccagtaacgttatacgatgtcgcagagtatgccggtgtctcttatcagaccgtttcccgcgtggtgaa

ccaggccagccacgtttctgcgaaaacgcgggaaaaagtggaagcggcgatggcggagctgaattacattcccaaccgc

gtggcacaacaactggcgggcaaacagtcgttgctgattggcgttgccacctccagtctggccctgcacgcgccgtcgc

aaattgtcgcggcgattaaatctcgcgccgatcaactgggtgccagcgtggtggtgtcgatggtagaacgaagcggcgt

cgaagcctgtaaagcggcggtgcacaatcttctcgcgcaacgcgtcagtgggctgatcattaactatccgctggatgac

caggatgccattgctgtggaagctgcctgcactaatgttccggcgttatttcttgatgtctctgaccagacacccatca

acagtattattttctcccatgaagacggtacgcgactgggcgtggagcatctggtcgcattgggtcaccagcaaatcgc

gctgttagcgggcccattaagttctgtctcggcgcgtctgcgtctggctggctggcataaatatctcactcgcaatcaa

attcagccgatagcggaacgggaaggcgactggagtgccatgtccggttttcaacaaaccatgcaaatgctgaatgagg

gcatcgttcccactgcgatgctggttgccaacgatcagatggcgctgggcgcaatgcgcgccattaccgagtccgggct

gcgcgttggtgcggatatctcggtagtgggatacgacgataccgaagacagctcatgttatatcccgccgttaaccacc

atcaaacaggattttcgcctgctggggcaaaccagcgtggaccgcttgctgcaactctctcagggccaggcggtgaagg

gcaatcagctgttgcccgtctcactggtgaaaagaaaaaccaccctggcgcccaatacgcaaaccgcctctccccgcgc

gttggccgattcattaatgcagctggcacgacaggtttcccgactggaaagcgggcagtgagcgcaacgcaattaatgt

aagttagcgcgaattgatctggtttgacagcttatcatcgactgcacggtgcaccaatgcttctggcgtcaggcagcca

tcggaagctgtggtatggctgtgcaggtcgtaaatcactgcataattcgtgtcgctcaaggcgcactcccgttctggat

aatgttttttgcgccgacatcataacggttctggcaaatattctgaaatgagctgttgacaattaatcatccggctcgt

ataatgtgtggaattgtgagcggataacaatttcagaattcaaaagatcttttaagaaggagatatacatATGAAACTT

TTAAAAGTAGCAGCAATTGCAGCAATCGTATTCTCCGGTAGCGCTCTGGCAGGTGTT GTTCCTCAGTACGGCGGCGGCG

GTAACCACGGTGGTGGCGGTAATAATAGCGGCCCAAATTCTGAGCTGAACATTTACC AGTACGGTGGCGGTAACTCTGC

ACTTGCTCTGCAAACTGATGCCCGTAACTCTGACTTGACTATTACCCAGCATGGCGG CGGTAATGGTGCAGATGTTGGT

CAGGGCTCAGATGACAGCTCAATCGATCTGACCCAACGTGGCTTCGGTAACAGCGC TACTCTTGATCAGTGGAACGGCA

AAAATTCTGAAATGACGGTTAAACAGTTCGGTGGTGGCAACGGTGCTGCAGTTGAC CAGACTGCATCTAACTCCTCCGT

CAACGTGACTCAGGTTGGCTTTGGTAACAACGCGACCGCTCATCAGTACGGTGGTGG TAGTGGTGGCGGCAGTGGTGGC

GGTAGCGGCGGTGGCTCCGGTGGCGGTTCTGGCGGCGGTTCTGGTGGTGGTTCTGGC GGTGGCTCAGGTGGGGGTTCCG

GCGGCGGTAGCGGCGGTGGATCTGGCGGCGGCTCTGAAGAATATGTGGGCCTGAGC GCGAACCAGTGCGCGGTGCCGGC

GAAAGATCGCGTGGATTGCGGCTATCCGCATGTGACCCCGAAAGAATGCAACAACC GCGGCTGCTGCTTTGATAGCCGC

ATTCCGGGCGTGCCGTGGTGCTTTAAACCGCTGCAGGAAGCGGAATGCACCTTTTGA CTCGAGtaaggatctccaggca

tcaaataaaacgaaaggctcagtcgaaagactgggcctttcgttttatctgttgtttgtcggtgaacgctctctactag

agtcacactggctcaccttcgggtgggcctttctgcgtttatacctagggcgttcggctgcggcgagcggtatcagctc

actcaaaggcggtaatacggttatccacagaatcaggggataacgcaggaaagaacatgtgagcaaaaggccagcaaaa

ggccaggaaccgtaaaaaggccgcgttgctggcgtttttccataggctccgcccccctgacgagcatcacaaaaatcga

cgctcaagtcagaggtggcgaaacccgacaggactataaagataccaggcgtttccccctggaagctccctcgtgcgct

ctcctgttccgaccctgccgcttaccggatacctgtccgcctttctcccttcgggaagcgtggcgctttctcatagctc

acgctgtaggtatctcagttcggtgtaggtcgttcgctccaagctgggctgtgtgcacgaaccccccgttcagcccgac

cgctgcgccttatccggtaactatcgtcttgagtccaacccggtaagacacgacttatcgccactggcagcagccactg

gtaacaggattagcagagcgaggtatgtaggcggtgctacagagttcttgaagtggtggcctaactacggctacactag

aaggacagtatttggtatctgcgctctgctgaagccagttaccttcggaaaaagagttggtagctcttgatccggcaaa

caaaccaccgctggtagcggtggtttttttgtttgcaagcagcagattacgcgcagaaaaaaaggatctcaagaagatc

ctttgatcttttctacggggtctgacgctcagtggaacgaaaactcacgttaagggattttggtcatgactagtgcttg

gattctcaccaataaaaaacgcccggcggcaaccgagcgttctgaacaaatccagatggagttctgaggtcattactgg

atctatcaacaggagtccaagcgagctcgtaaacttggtctgacagttacattggaaaacgttcttcggggcgaaaact

ctcaaggatcttaccgctgttgagatccagttcgatgtaacccactcgtgcacccaactgatcttcagcatcttttact

ttcaccagcgtttctgggtgagcaaaaacaggaaggcaaaatgccgcaaaaaagggaataagggcgacacggaaatgtt

gaatactcatactcttcctttttcaatattattgaagcatttatcagggttattgtctcatgagcggatacatatttga

atgtatttagaaaaataaacaaataggggttccgcgcacatttccccgaaaagtgccacct
